# SARM regulates cell apoptosis and inflammation during *Toxoplasma gondii* infection through a multistep mechanism

**DOI:** 10.1186/s13071-025-06721-2

**Published:** 2025-03-12

**Authors:** Shumin Gao, Min Gao, Huanhui Du, Lingyu Li, Xudian An, Yongyu Shi, Xiaoyan Wang, Hua Cong, Bing Han, Chunxue Zhou, Huaiyu Zhou

**Affiliations:** 1 Department of Pathogen Biology, School of Basic Medical Sciences, Cheeloo Medical College, Shandong University, Jinan, Shandong People’s Republic of China; 2 Department of Immunology, School of Basic Medical Sciences, Cheeloo Medical College, Shandong University, Jinan, Shandong People’s Republic of China; 3 Present Address: National Institute On Drug Dependence, Peking University, Beijing, People’s Republic of China

**Keywords:** SARM, *Toxoplasma gondii*, Apoptosis, Inflammation, Infection

## Abstract

**Background:**

The sterile alpha and HEAT/Armadillo motif (SARM) is the fifth Toll-like receptor (TLR) adaptor protein containing the Toll/interleukin-1 receptor (TIR) domain, which is highly enriched in the brain. *Toxoplasma gondii* (*T. gondii*) is an obligate intracellular parasitic protozoan that causes zoonotic toxoplasmosis, resulting in threats to human health, such as brain damage. Previous studies have shown that SARM plays crucial roles in cell death and triggers specific transcription programs of innate immunity in response to cell stress, viral, and bacterial infections. However, whether SARM is involved in *T. gondii* infection remains unclear.

**Methods:**

In this report, quantitative real-time polymerase chain reaction (qPCR), western blot, flow cytometry, ethynyldeoxyuridine (EdU) assay, and enzyme-linked immunosorbent assay (ELISA) were used to explore the relationship between SARM and *T. gondii*.

**Results:**

Here, we showed that *T. gondii* infection increased the expression of SARM in vitro and in vivo. SARM induced cell apoptosis during *T. gondii* infection, activating the mitochondrial apoptotic pathway, the endoplasmic reticulum stress (ER) pathway, and the mitogen-activated protein kinase (MAPK) signaling pathway, and prompting the production of reactive oxygen species (ROS). Furthermore, SARM participated in the regulation of the inflammatory response through the nod-like receptor pyrin domain 3 (NLRP3) inflammasome signaling pathway during *T. gondii *in vitro infection.

**Conclusions:**

These results elucidate the relationship between SARM and *T. gondii* infection, suggesting that SARM may represent a potential target for *T. gondii* control.

**Graphical Abstract:**

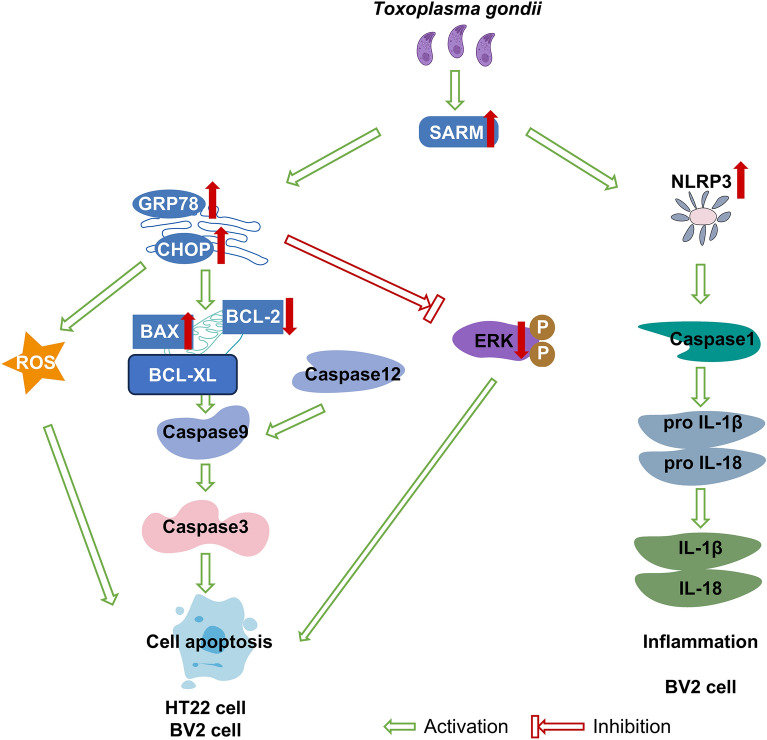

**Supplementary Information:**

The online version contains supplementary material available at 10.1186/s13071-025-06721-2.

## Background

*Toxoplasma gondii* (*T. gondii*) is a worldwide obligate intracellular parasite that infects nearly all warm-blooded vertebrates [1, 2]. Approximately one-third of the world’s population is infected with *T. gondii*, and most of them are asymptomatic [3]. However, immunocompromised individuals, such as those with the acquired immunodeficiency syndrome or those receiving immunosuppression after organ transplantation, tend to develop retinal choroid plexus, localized pneumonia, acute respiratory failure, hemodynamic abnormalities, epileptic paresthesia, dyskinesia, and encephalitis following *T. gondii* infection [4, 5]. In addition, if a woman is infected with *T. gondii* in early pregnancy or during pregnancy, the parasite can cross the placental barrier, leading to miscarriage or birth defects [6]. So far, there is no effective agent to treat toxoplasmosis.

The innate immunity system is the first line of host defense against pathogens. It mainly recognizes pathogen-specific conserved structures, which are called pathogen-associated molecular patterns. Toll-like receptors (TLRs) and their activated signaling pathways play essential roles in the infection of host anti-parasites [7], for instance, TLR11 recognizes *T. gondii*’s unconventional actin-binding protein profiling [8]. Additionally, TLR2, TLR7, and TLR9 are all possible candidates for innate immune sensors that could be engaged in human defense against *T. gondii* [8]. With regards to nod-like receptors (NLRs), nod-like receptor pyrin domain 1 (NLRP1) was the first sensor reported to be involved in *T. gondii* pathogenesis [9, 10]. NLRP3 was also thought to take part in *T. gondii* sensing by inducing interleukin 18 (IL-18), which was shown to be important for restricting parasite infection [11]. Besides, NLRP3, apoptosis-associated speck-like protein containing a caspase recruitment domain (CARD), cysteinyl aspartate specific proteinase (Caspase1), and IL-1 receptor (IL-1R) have protective roles in response to *T. gondii* infection [11]. However, the mechanisms of NLRP3 activation by *T. gondii* have not been elucidated.

Sterile alpha and HEAT/Armadillo motif (SARM) is the fifth member of the TLR adaptor family and is highly conserved across many species including worms, *Drosophila*, and mammals [12]. In 2006, Carty et al. characterized SARM as a negative regulator of TLR signaling [13]. Recently, several examples show that SARM has significant functions in antiviral innate immune responses and mediating apoptosis [14, 15]. Hou et al. found that SARM^−/−^ mice were more susceptible to West Nile virus than wild-type (WT) mice, and the expression of tumor necrosis factor alpha (TNF-α) in the brainstem of mice was regulated by SARM, indicating that SARM played a role in immune protection [16]. Bunyavirus can upregulate the expression of SARM protein and recruit SARM to mitochondria, causing neuronal apoptosis. SARM^−/−^ mice infected with bunyavirus showed a higher survival rate, which indicates that SARM mediates the harmful immune response of the mammalian host [17]. Additionally, Carty et al. found that SARM negatively regulated NLRP3 to reduce the secretion of IL-1β [18]. Further evidence supporting the role of SARM in mediating the innate immune response found that SARM is required for proinflammatory cytokines expression in response to vesicular stomatitis virus infection in the brain. The expression of IL-6, TNF, macrophage inflammatory protein-1 alpha (MIP-1α), monocyte chemoattractant protein-1 (MCP-1), regulated on activation, normal T cell expressed, and secreted (RANTES), interferon α (IFNα), and IFNβ all required SARM [19].

*T. gondii* infection can both inhibit and induce host cell apoptosis to improve its survival [20, 21]. Recently, Dincel et al. found that the ME49 chronic low-virulence *T. gondii* strain induced severe neurodegeneration in mice with *T. gondii* encephalitis, and this was accompanied by high expression of apoptotic mediators a disintegrin and metalloproteinase with a thrombospondin type 1 motif, member 13 (ADAMTS-13), Caspase3, Caspase8, Caspase9, TNF receptor 1 (TNFR1), and nitric oxide (NO) [22, 23]. An et al. demonstrated that the *T. gondii* virulence factor polymorphic rhoptry protein 18 (ROP18) activated endoplasmic reticulum (ER) stress-mediated apoptosis by phosphorylating the ER-associated protein reticulon 1-C (RTN1-C), thereby inducing neuronal apoptosis [24]. However, the mechanism by which SARM acts during *T. gondii* infection remains to be fully elucidated.

Here, we proved that SARM played key roles in *T. gondii* infection by promoting intercellular reactive oxygen species (ROS) production, activating both mitochondrial and ER-induced apoptosis, and inhibiting the mitogen-activated protein kinase (MAPK) signaling pathway. Furthermore, *T. gondii* infection regulated the NLRP3 inflammasome signaling pathway via SARM. Our research explored the role of SARM in *T. gondii* infection, providing a novel target and a new idea for the prevention and treatment of *T. gondii*.

## Methods

### Parasite, cell, and animal

The highly virulent tachyzoites of the *T. gondii* RH strain were preserved in our laboratory at the School of Basic Medical Sciences, Shandong University, People’s Republic of China. Tachyzoites were maintained by serial passages in human foreskin fibroblast (HFF) cells. In brief, tachyzoites were added to HFF cells, which were at a density of 80–90%. About 3 days post-infection (dpi), a large number of *T. gondii* tachyzoites can be observed in the culture flask. To purify tachyzoites, parasites that egressed naturally from cells were collected and passed through a 27-gauge needle, and purified by passage through five μm pore filters. BV2 cell line, HT22 cell line and HFF cell line were cultured in Dulbecco’s modified Eagle’s medium supplemented with 10% fetal bovine serum and 100 IU/mL penicillin and 100 μg/mL streptomycin at 37 ℃ in a humidified chamber with 5% CO_2_. Specific-pathogen-free male Kunming (KM) mice aged 6–8 weeks were purchased from the Animal Center of Shandong University, People’s Republic of China. All the mice were allowed access to food and water ad libitum and were handled in strict accordance with good animal practice as per the animal ethics procedures.

### Antibodies

The primary antibodies (Abs) of SARM (cat. no.: ab17812), BCl-2 (cat. no.: ab182858), BCL-XL (cat. no.: ab32370), Caspase1 (cat. no.: ab179515), Caspase3 (cat. no.: ab184787), phospho-extracellular regulated protein kinases (ERK) (cat. no.: ab201015), ERK (cat. no.: ab184699), NLRP3 (cat. no.: ab263899), IL-18 (cat. no.: ab207323), IL-1β (cat. no.: ab234437), GRP78 (cat. no.: ab21685), and CHOP (cat. no.: ab11419) were purchased from Abcam (Cambridge, UK). The primary Abs of Caspase9 (cat. no.: 9504) and Caspase12 (cat. no.: 2202) were purchased from Cell Signaling Technology (Boston, the United States). The BAX primary antibody (cat. no.: A5131) was purchased from Selleckchem (Houston, TX, USA). β-Tubulin monoclonal antibody (cat. no.: AB0039) was purchased from Abways Technology (Shanghai, China). GAPDH monoclonal antibody (cat. no.: 60004-1-Ig) and β-actin monoclonal antibody (cat. no.: 66009-1-Ig) were purchased from Proteintech Group (Wuhan, China). The secondary HRP-conjugated antibodies were purchased from Proteintech Group (Wuhan, China).

### *T. gondii* infection

A total of 10,000 *T. gondii* tachyzoites were inoculated via intraperitoneal injection into 6- to 8-week-old male KM mice, with five mice in each experimental group. At 0, 1, 2, 3, 4, 5, 6, and 7 dpi, the mice were decapitated following carbon dioxide exposure, and tissues were harvested. Firstly, parasites were used to infect cells in vitro at a multiplicity of infection (MOI) of one, two, three, four, and five, respectively for 24 h. Subsequently, the cells infected at an MOI of two were selected for further tests.

### Lentivirus transduction

A recombinant lentivirus containing the SARM gene was designed by Shanghai Genechem, People’s Republic of China. HT22 cells were seeded into a 6-well plate at 3 × 10^4^ cells per well and cultured at 37 ℃ in a 5% CO_2_ incubator for 24 h. Then, HT22 cells were infected with lentivirus at an MOI of five, and the medium was changed after an additional 8 h of culture. After 3–4 days, puromycin was added to select for stable cell lines.

### Cell transfection

The murine SARM gene was cloned into pRK5 plasmid. Then pRK5-SARM plasmids were used for transfection experiments. HT22 cells or BV2 cells were seeded in 6-well plates at a density of 4 × 10^5^ cells/well and grown overnight before transfection. Each transfection mixture, containing 4 μg of plasmid in 400 μL of Opti-MEM and 6 μL of Lipofectamine Plus Reagent 2000 (Invitrogen, Carlsbad, CA, USA) was incubated at room temperature for 20 min. The transfection mixture was then added to the cells. After 6 h, Opti-MEM was replaced with the complete medium. After 24 h post-transfection (hpi), cells were infected with *T. gondii* at an MOI of two.

### Ethynyldeoxyuridine (EdU) assay

HT22 cells or BV2 cells were seeded in 6-well plates at a density of 4 × 10^5^ cells/well and grown overnight before transfection. Cells were transfected with pRK5-SARM plasmid; 24 hpi, the cells were digested and seeded in 96-well plates at a density of 3 × 10^3^ cells/well with three multiple holes in each group and cultured in a 5% CO_2_ incubator at 37 ℃ for 24 h. Then, the cell proliferation was evaluated using the EdU detection kit (RiboBio, Guangzhou, China) according to the manufacturer’s instructions. The EdU incorporation rate was calculated as the ratio of the number of EdU-incorporated cells to the number of Hoechst 33342-staining cells.

### Cellular apoptosis assay

The apoptosis ratio was analyzed using the Annexin V-PE Apoptosis Detection Kit (BD Biosciences, New Jersey, USA). Annexin V identifies surface-exposed phosphatidylserine, and 7-AAD is retained in late apoptotic cells. The samples were analyzed by flow cytometry (Beckman CytoFLEX, Brea, CA, USA). Cells were discriminated into viable cells, necrotic cells, and apoptotic cells using Beckman CytoFLEX software (Beckman CytoFLEX, Hercules, CA, USA), and then the percentages of apoptotic cells from each group were compared.

### Western blot

Cells were washed with ice-cold phosphate buffer saline (PBS) twice and then RIPA lysis buffer (Beyotime Biotechnology, Shanghai, China) was added, which contained protease inhibitor cocktail (Bimake, Houston, TX, USA) and phosphatase inhibitor cocktail (Bimake, Houston, TX, USA). The protein concentration was measured using the BCA protein assay kit (Beyotime Biotechnology, Shanghai, China) to quantify the proteins. The samples were heated at 100 ℃ for 5 min. Subsequently, samples with equal protein content were loaded on sodium dodecyl-sulfate polyacrylamide gel electrophoresis (SDS-PAGE) gels and electrophoresed, then transferred onto polyvinylidene fluoride (PVDF) membranes. The PVDF membranes were blocked for 2 h in 5% bovine serum albumin (BSA) at room temperature and probed overnight with primary Abs at 1:1000 at 4 ℃. The next day, membranes were incubated with secondary HRP-conjugated Abs at 1:10000 for 1 h at room temperature. Finally, the blots were detected using a western blot analysis system.

### Quantitative real-time polymerase chain reaction (qPCR)

Total RNA was extracted using AG RNAex Pro Reagent (Accurate Biotechnology Co., Ltd., Hunan, China), and the extracted RNA was reverse-transcribed into cDNA using the HiScriptII RT SuperMix for qPCR (Vazyme Biotech Co. Ltd., Nanjing, China). SYBR green dye (Accurate Biotechnology Co., Ltd., Hunan, China) was used to measure real-time PCR amplifications. All reactions were carried out in a 20 µL reaction mixture with following steps: 95 ℃ for 30 s, 40 cycles of 95 ℃ for 5 s, 60 ℃ for 30 s, melting curve at 95 ℃ for 15 s, 60 ℃ for 60 s, and 95 ℃ for 15 s. The mRNA levels of each sample for each gene were normalized against those of β-actin mRNA. All primers were synthesized by Genewiz (Tianjin, China), and their sequences were as follows: β-actin forward: 5′-GGCTGTATTCCCCTCCATCG-3′; β-actin reverse: 5′-CCAGTTGGTAACAATGCCATGT-3′; SARM forward: 5′-TGCAGGACCATGACTGCAAG-3′; and SARM reverse: 5′-TCATGGGACCATTTGATGCCG-3′. The ΔΔCT method was used to determine differences in gene expression levels after normalization to the arithmetic mean of β-actin. All data are presented as the mean ± standard deviation (SD) of three independent experiments.

### ROS assay

HT22 cells or BV2 cells were transfected with pRK5-SARM plasmid and followed by *T. gondii* infection for 24 h. The cells were digested and collected. 2′-7′-Dichlorodihydrofluorescein diacetate (DCFH-DA; Beyotime Biotechnology, Shanghai, China) was diluted with serum-free culture medium at a ratio of 1:1000 to a final concentration of 10 µM. Cells were re-suspended in the diluted DCFH-DA and the cell concentration was adjusted to 1–20 × 10^6^ cells/mL. Then the cells were incubated in a 37 °C incubator for 20 min, and mixed upside down every 3–5 min to ensure full contact between the probe and the cells. Cells were washed three times with serum-free cell culture medium to fully remove the DCFH-DA that had not entered the cells. The results were recorded by performing flow cytometry detection.

### Enzyme-linked immunosorbent assay (ELISA)

To determine the effects of SARM on cytokine release, HT22 cells or BV2 cells that were overexpressing SARM were infected with *T. gondii* for 24 h. The levels of IL-18 secreted by the cells were quantified in 24 h supernatants by using mouse IL-18 ELISA kit (MultiSciences (Lianke) Biotech Co., Ltd., Hangzhou, China) according to the manufacturer’s instructions.

### Statistical analysis

All statistical analyses were done using GraphPad Prism 8. The data were expressed as mean ± SD. Significance was determined by Student’s t-test or one-way ANOVA. Statistical differences are represented by *P < 0.05, **P < 0.01, and ***P < 0.001; ns means not significant. Each experiment was repeated at least three times as biological replicates, unless otherwise indicated.

## Results

### SARM is highly expressed in the nervous system

The expression pattern of SARM was verified through western blot analysis of protein from various organs in mice (Additional file 1: Fig. S1A). We found that SARM is highly expressed in the brain and lung, demonstrating a similarity to the pattern observed in humans (Additional file 1: Fig. S1B). Additionally, we found that SARM gene expression existed in all types of cells in the brain (Additional file 1: Figs. S1C and S1D), including neurons and microglia. Thus, we speculated that SARM might play an important role in the central nervous system.

### The expression of SARM is upregulated after *T. gondii* infection in vivo and in vitro

**Fig. 1:**
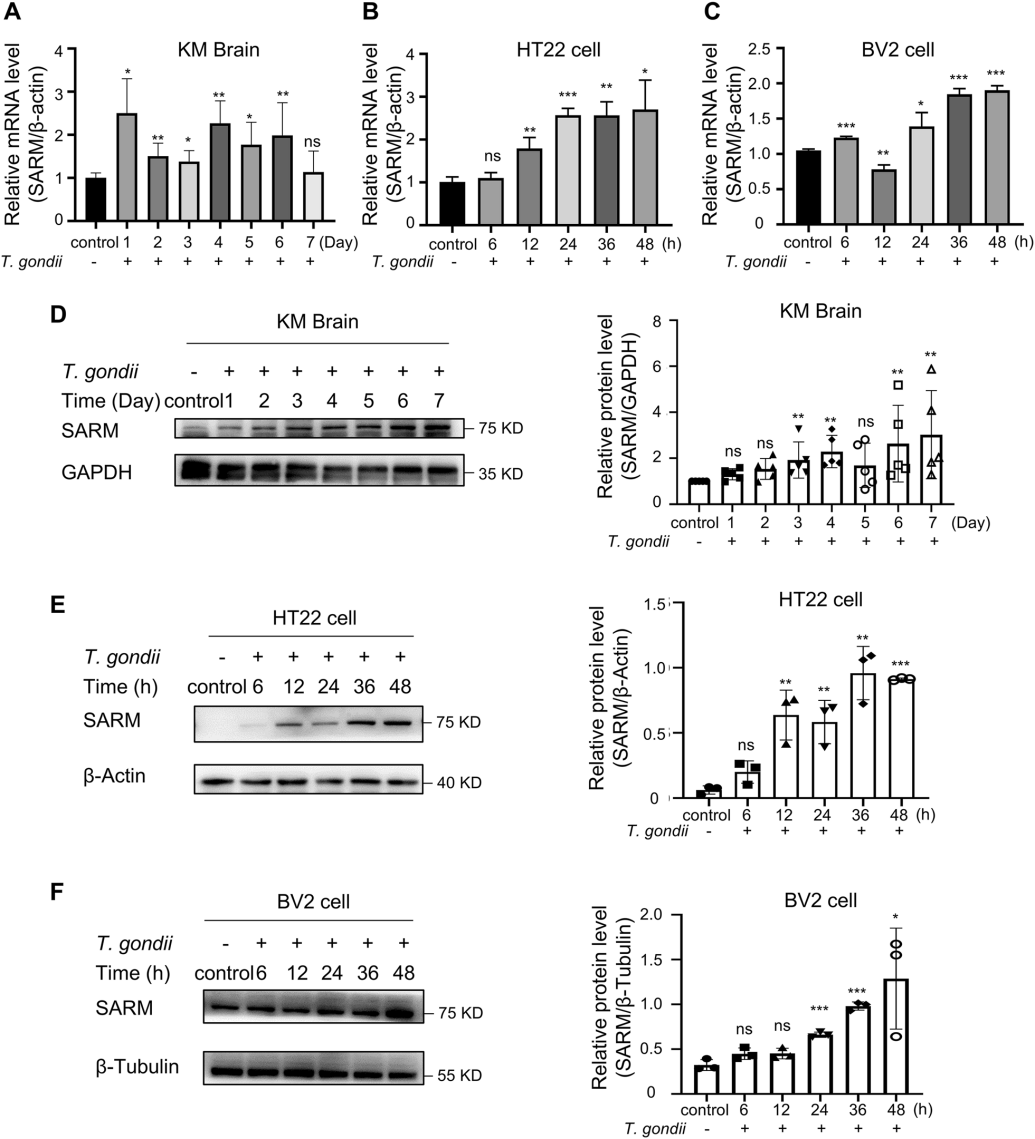
The increased expression of SARM after *T. gondii *in vivo and in vitro. **A**-**C**, The qPCR for SARM mRNA was performed on *T. gondii*-infected KM mouse brains and age-matched noninfected brain homogenates from KM mouse brains in vivo. (**A**), *T. gondii*-infected HT22 cells and noninfected HT22 cells in vitro (**B**), *T. gondii*-infected BV2 cells and noninfected BV2 cells in vitro (**C**). Relative expression was normalized to actin expression. **A** *n* = 5 mice per group. **B**, **C** *n* = 3 per group. **D**–**F**, Western blot detection of SARM and GAPDH expression in mouse brains infected and noninfected with *T. gondii* in vivo (**D**), SARM and Tubulin expression in *T. gondii*-infected HT22 cells and noninfected HT22 cells in vitro at an MOI of 2 (**E**), and *T. gondii*-infected BV2 cells and noninfected BV2 cells at an MOI of 2 (**F**). Densitometric quantification of the SARM western blot was performed. The qPCR and western blot results represented at least three independent experiments. **D**, *n* = 5 mice per group. **E** and **F**, *n* = 3 per group. Data were shown as mean ± SD. ns, not significant, compared with control group; *P < 0.05, compared with control group; **P < 0.01, compared with control group; ***P < 0.001, compared with control group

To determine whether the expression of SARM is altered after *T. gondii* infection in vivo, we performed qPCR for mRNA and western blot for protein isolated from brains of KM mice infected with *T. gondii* RH strain. The qPCR result indicated that SARM mRNA was significantly augmented in *T. gondii*-infected mouse brains from the first to the sixth dpi (Fig. 1A). Then, we performed western blot analyses to assess the SARM protein levels in KM mouse brains infected with *T. gondii*. Meanwhile, we observed that the SARM protein level was significantly increased in *T. gondii*-infected KM mouse brains (Fig. 1D). Subsequently, we chose different MOIs to treat HT22 cells and BV2 cells. We found that *T. gondii* could increase the SARM expression relatively high at an MOI of two (Additional file 4: Fig. S4). In addition, we detected the mRNA and protein expression of SARM in HT22 cells and BV2 cells infected with *T. gondii* at an MOI of two. The mRNA and protein expression of SARM were significantly increased in *T. gondii*-infected HT22 cells at 12 hpi to 48 hpi compared with the control. Strikingly, the SARM expression did not show a very significant change at 6 hpi, even though there was a slight decrease at protein expression level (Fig. 1B, E). SARM mRNA expression was significantly increased at 6 hpi, 24 hpi, and 48 hpi but decreased at 12 hpi in BV2 cells infected with *T. gondii* (Fig. 1C). The protein expression of SARM in BV2 cells infected with *T. gondii* increased from 24 hpi to 48 hpi, and the difference was statistically significant (Fig. 1F). Consequently, our results indicated that *T. gondii* infection resulted in the upregulation of SARM mRNA and protein expressions.

### SARM contributed to *T. gondii*-mediated cell apoptosis

**Fig. 2:**
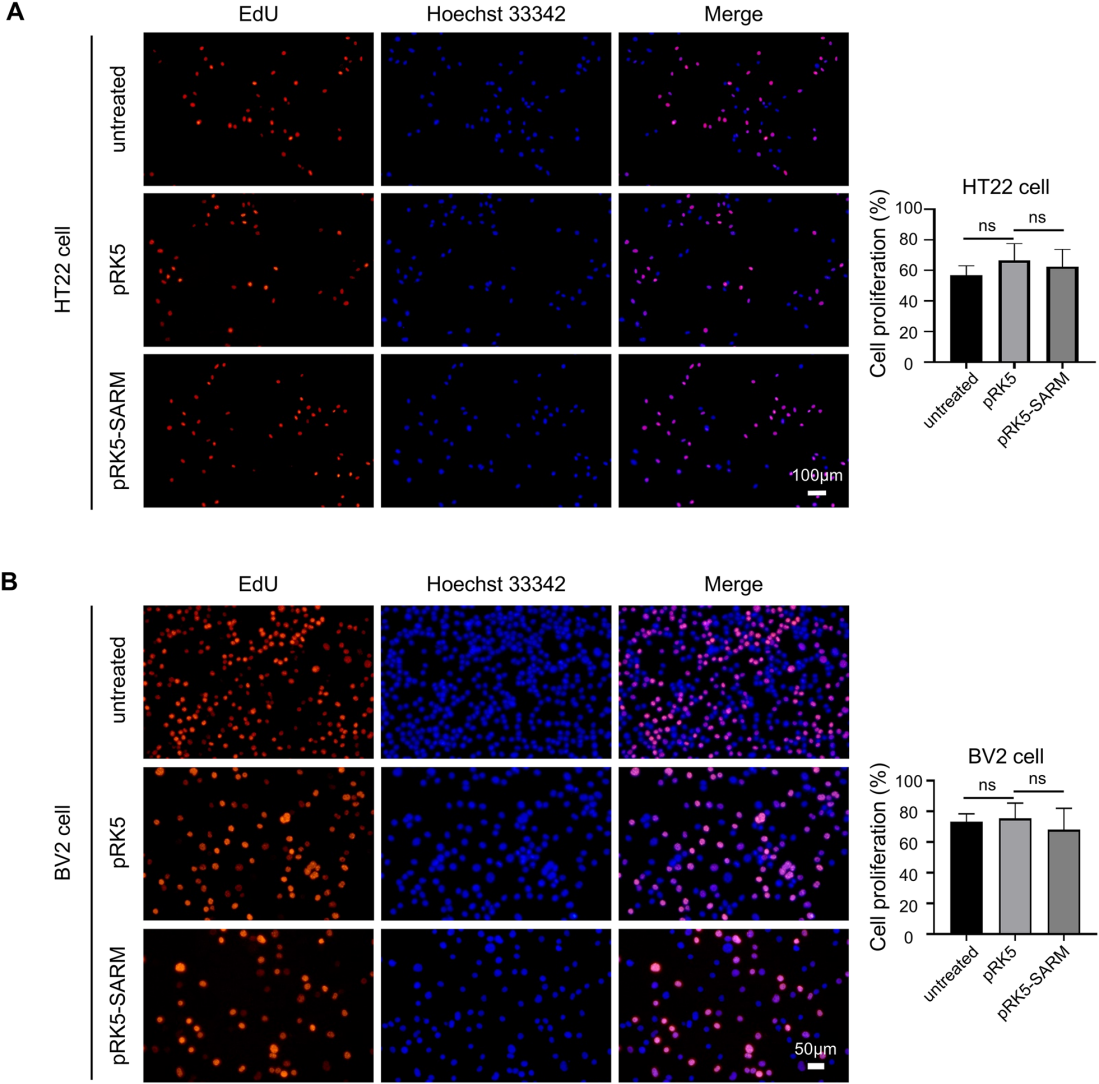
SARM did not affect cell proliferation. HT22 and BV2 cells were transiently transfected with 4 μg of SARM plasmids. At 24 hpi, cells were seeded into 96-well plates and cultured for 24 h. The EdU incorporation rate was calculated as the ratio of the number of EdU-incorporated cells to Hoechst 33342-stained cells. **A**, Transient overexpression of SARM in HT22 cells did not affect cell proliferation, scale bar represents 100 μm. *n* = 5 per group. **B**, Transient overexpression of SARM in BV2 cells did not affect cell proliferation, scale bar represents 50 μm. *n* = 3 per group. Data were shown as mean ± SD. ns, not significant

**Fig. 3:**
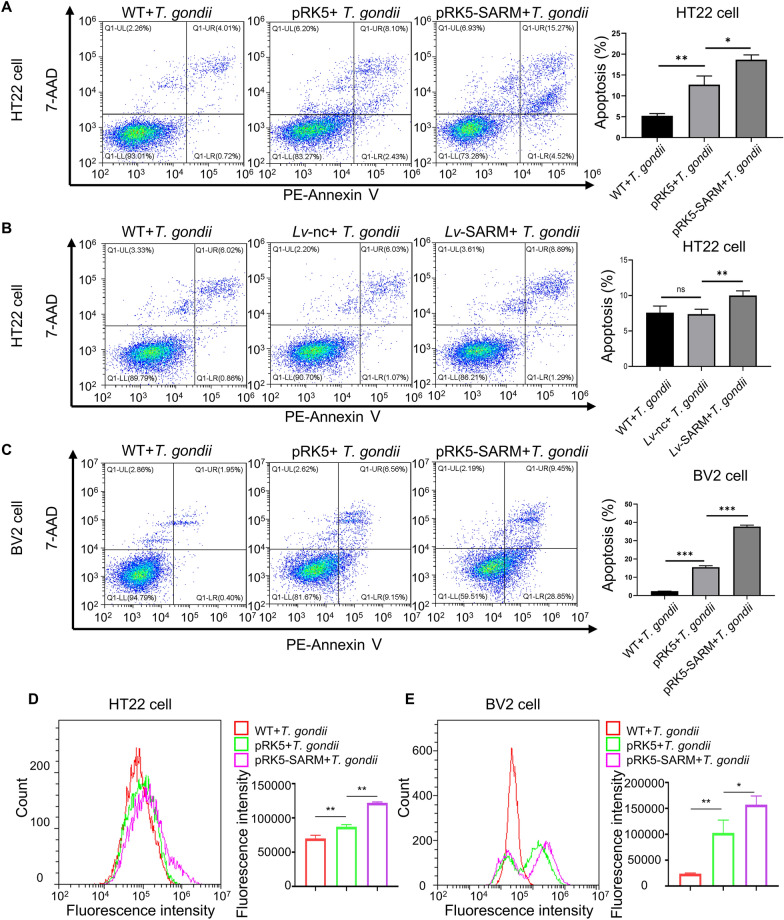
Induction of cell apoptosis and ROS by SARM in HT22 and BV2 cells. **A**–**C**, SARM overexpression accelerated *T. gondii*-mediated cell apoptosis. HT22 cells were transiently transfected with 4 μg of SARM plasmids. After 24 h of SARM overexpression, the cells were co-cultured with *T. gondii* tachyzoites for 24 h, and cell apoptosis was measured by flow cytometry (**A**). HT22 cells were stably transfected with SARM and co-cultured with *T. gondii* tachyzoites for 24 h, cell apoptosis was measured by flow cytometry (**B**). BV2 cells were transiently transfected with 4 μg of SARM plasmids. After 24 h of SARM overexpression, the cells were co-cultured with *T. gondii* tachyzoites for 24 h, cell apoptosis was measured by flow cytometry (**C**). **D** and **E**, SARM overexpression generated *T. gondii*-mediated ROS. HT22 cells were transiently transfected with 4 μg of SARM plasmids after 24 h of overexpression of SARM and co-cultured with *T. gondii* tachyzoites for 24 h, and intracellular ROS was measured by flow cytometry (**D**). BV2 cells were transiently transfected with 4 μg of SARM plasmids after 24 h of overexpression of SARM and co-cultured with *T. gondii* tachyzoites for 24 h, intracellular ROS was measured by flow cytometry (**E**). *n* = 3 per group. Data are shown as mean ± SD. *P < 0.05; **P < 0.01, ***P < 0.001

To explore the function of SARM, we constructed a SARM overexpression plasmid (Additional file 2: Fig. S2), SARM overexpression virus, and verified the expression of the SARM overexpression plasmids in HT22 and BV2 cells and the SARM overexpression virus in HT22 cells (Additional file 3: Fig. S3). The EdU assay was used to determine the effect of SARM overexpression on cell proliferation. The results indicated that the transient overexpression of SARM in HT22 and BV2 cells had no effect on cell proliferation (Fig. 2). Then, the role of SARM in *T. gondii*-mediated cell apoptosis was assessed by flow cytometry in HT22 and BV2 cells transfected with SARM overexpression plasmids. The results demonstrated that both transient transfection and stable transfection of SARM in HT22 cells upregulated apoptosis after *T. gondii* infection (Fig. 3A, B). Similarly, transient transfection of SARM in BV2 cells also upregulated cell apoptosis after *T. gondii* infection (Fig. 3C). Meanwhile, we observed an increasing trend of intracellular ROS in SARM-overexpressing HT22 and BV2 cells infected with *T. gondii* (Fig. 3D, E). Taken together, the results suggested SARM mediated cell apoptosis during *T. gondii* infection, possibly through a mechanism related to the production of ROS.

### SARM activated the mitochondrial apoptosis pathway

**Fig. 4:**
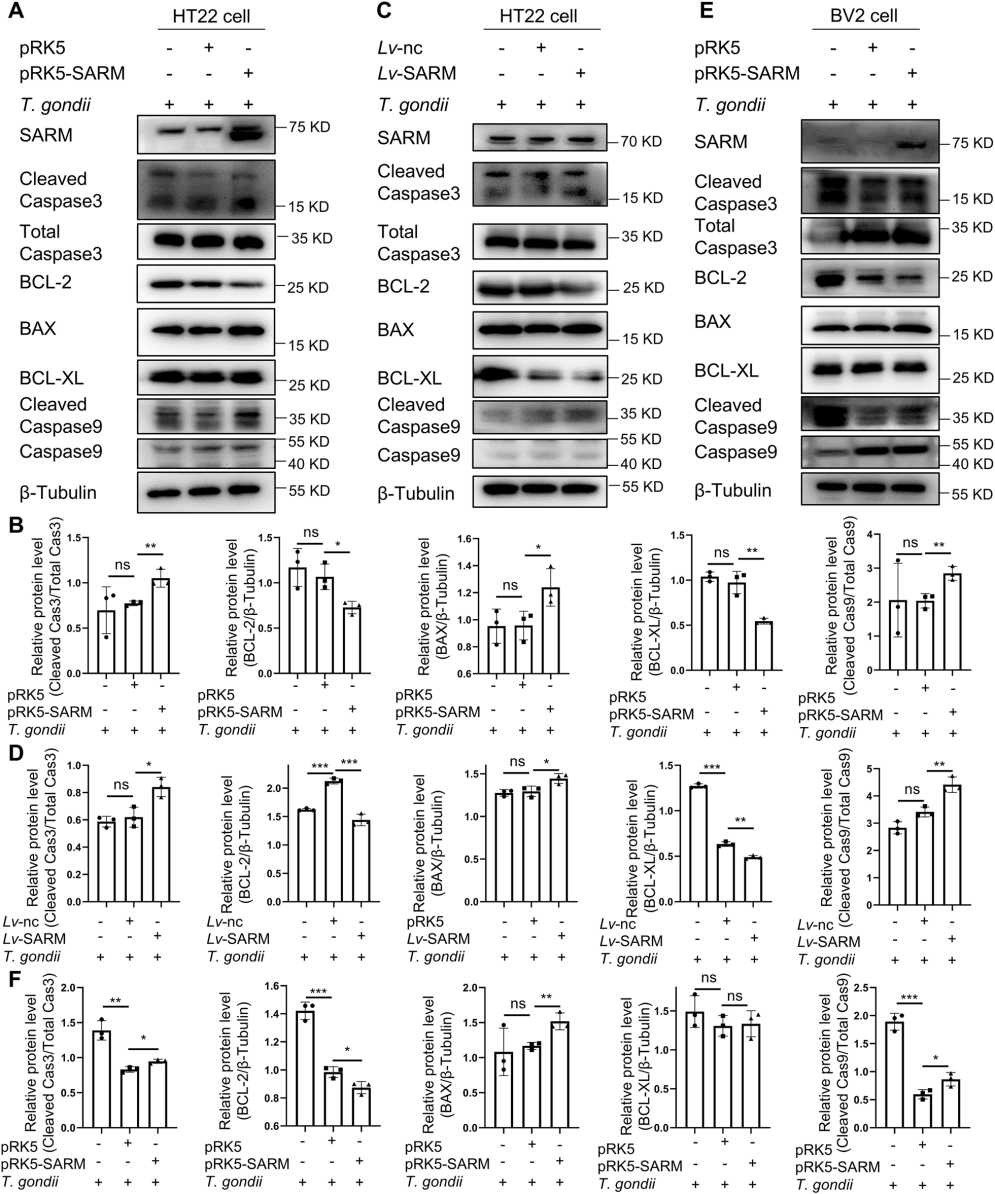
SARM mediated cell apoptosis via the mitochondrial apoptosis pathway. **A**, HT22 cells were transiently transfected with 4 μg of SARM plasmids after 24 h of overexpression of SARM and co-cultured with *T. gondii* tachyzoites for 24 h; mitochondrial apoptosis pathway-related proteins were examined by western blot. **B**, The chart shows the quantification of relative protein expression (**A**). **C**, HT22 cells were stably transfected with SARM and co-cultured with *T. gondii* tachyzoites for 24 h, and mitochondrial apoptosis pathway-related proteins were examined by western blot. **D**, The chart shows the quantification of relative protein expression (**C**). **E**, BV2 cells were transiently transfected with 4 μg of SARM plasmids after 24 h of overexpression of SARM and co-cultured with *T. gondii* tachyzoites for 24 h, and mitochondrial apoptosis pathway-related proteins were examined by western blot. **F**, The chart shows the quantification of relative protein expression of (**E**). *n* = 3 per group. Data are shown as mean ± SD. ns, not significant; *P < 0.05; **P < 0.01; ***P < 0.001

To further confirm that SARM-induced apoptosis was dependent on the mitochondrial apoptosis pathway, the protein expressions of B-cell lymphoma-2 (BCL-2), BCL2-associated X (BAX), cleaved Caspase9, cleaved Caspase3, and BCL-extra large (BCL-XL) was detected via western blot. Transient overexpression of SARM upregulated the expression of pro-apoptotic proteins BAX, cleaved Caspase9, and cleaved Caspase3 in HT22 and BV2 cells infected with *T. gondii*, and downregulated the expression of anti-apoptotic protein BCL-2. However, there was no significant change in the expression of the anti-apoptotic protein BCL-XL in BV2 cells infected with *T. gondii* (Fig. 4A, B, E, F). Similar results were observed in stable overexpression of SARM in HT22 cells (Fig. 4C, D). These data indicated SARM-mediated cell apoptosis during *T. gondii* infection was dependent on the mitochondrial apoptosis pathway.

### SARM executed cell apoptosis via endoplasmic reticulum (ER) stress-induced apoptosis

**Fig. 5:**
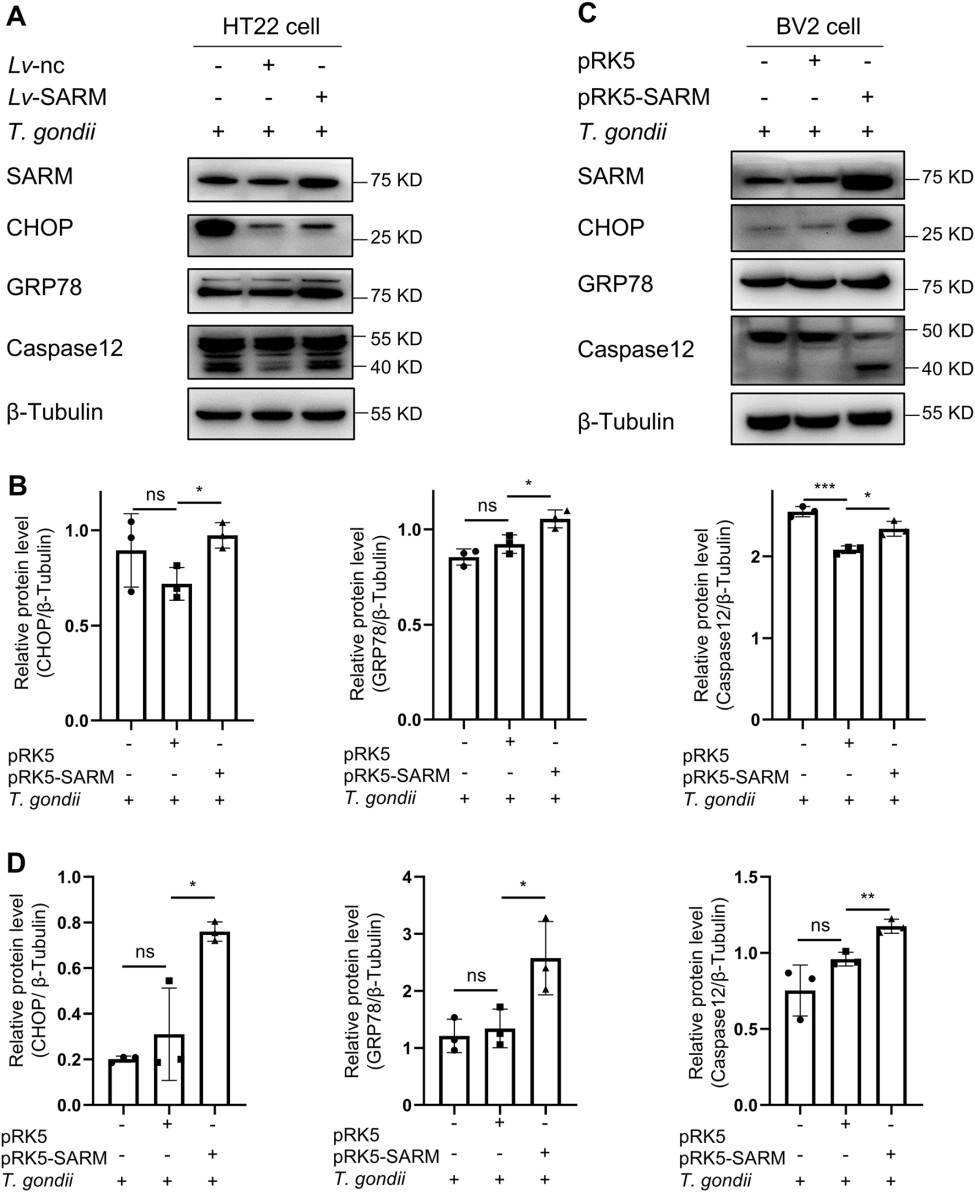
SARM mediated cell apoptosis via ER stress-induced pathway. **A**, HT22 cells were stably transfected with SARM and co-cultured with *T. gondii* tachyzoites for 24 h; ER stress-induced pathway-related proteins were examined by western blot. **B**, The chart shows the quantification of relative protein expression (**A**). **C**, BV2 cells were transiently transfected with 4 μg of SARM plasmids after 24 h of overexpression of SARM and co-cultured with *T. gondii* tachyzoites for 24 h, ER stress-induced pathway-related proteins were examined by western blot. **D**, The chart shows the quantification of relative protein expression (**B**). *n* = 3 group. Data are shown as mean ± SD. ns, not significant; *P < 0.05; **P < 0.01; ***P < 0.001

The previous results showed that SARM promoted cell apoptosis during *T. gondii* infection, but the specific mechanism was not clear. Therefore, we investigated the effect of SARM on the expression of proteins related to the apoptosis pathway induced by ER stress during *T. gondii* infection. Stable transfection of SARM in HT22 cells upregulated the protein expression of C/EBP homologous protein (CHOP), G-protein coupled receptor 78 (GRP78) and Caspase12 during *T. gondii* infection (Fig. 5A), while transient transfection of SARM in BV2 cells also upregulated the expression of CHOP, GRP78 and Caspase12 proteins during *T. gondii* infection (Fig. 5B). These data clearly demonstrated that SARM executed cell apoptosis via ER stress-induced apoptosis.

### SARM inhibited extracellular regulated protein kinases (ERK) phosphorylation

**Fig. 6:**
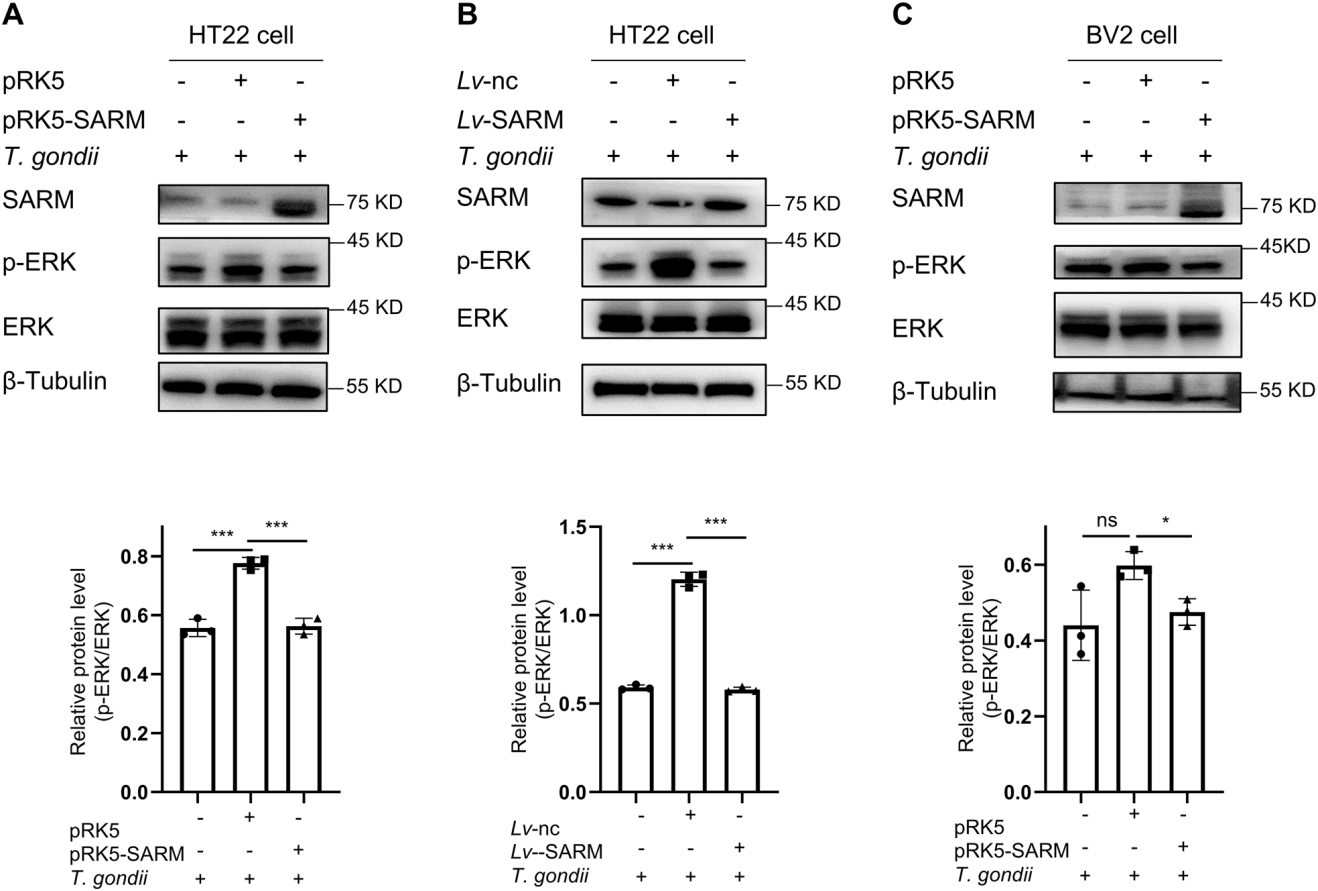
SARM inhibited ERK phosphorylation. **A**, HT22 cells were transiently transfected with 4 μg of SARM plasmids after 24 h of overexpression of SARM and co-cultured with *T. gondii* tachyzoites for 24 h, and ERK phosphorylation was detected via western blot. **B**, HT22 cells were stably transfected with SARM and co-cultured with *T. gondii* tachyzoites for 24 h, and ERK phosphorylation was detected via western blot. **C**, BV2 cells were transiently transfected with 4 μg of SARM plasmids after 24 h of overexpression of SARM and co-cultured with *T. gondii* tachyzoites for 24 h; ERK phosphorylation was detected via western blot. *n* = 3 per group. Data are shown as mean ± SD. ns, not significant; *P < 0.05; ***P < 0.001

We used western blot to examine the effect of SARM on the expression of MAPK-related proteins during *T. gondii* infection. The results indicated that when SARM was overexpressed by either transient or stable transfection, the p-ERK/ERK ratio was significantly decreased in HT22 cells infected with *T. gondii* (Fig. 6A, B). In addition, the p-ERK/ERK ratio was also significantly decreased after transient transfection of SARM in BV2 cells infected with *T. gondii* (Fig. 6C). These data strongly corroborated that SARM inhibited ERK phosphorylation.

### SARM tuned NLRP3 inflammasome signaling pathway

**Fig. 7:**
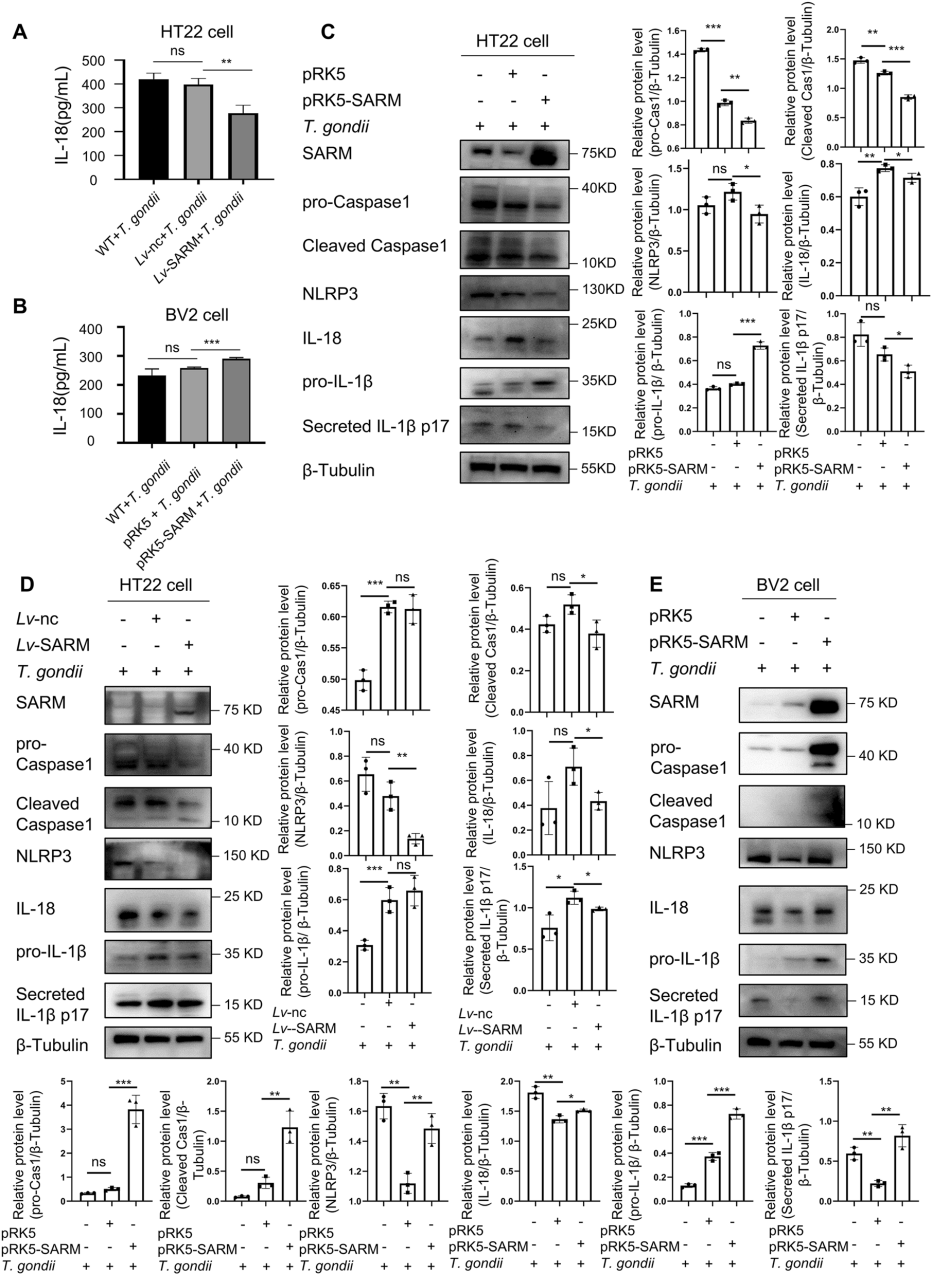
SARM tuned NLRP3 signal pathway. **A** and **B**, SARM regulated the secretion of IL-18 in HT22 cells and BV2cells. HT22 cells were stably transfected with SARM and co-cultured with *T. gondii* tachyzoites for 24 h, and IL-18 secretion was detected via ELISA (**A**). *n* = 4 per group. BV2 cells were transiently transfected with 4 μg of SARM plasmids after 24 h of overexpression of SARM and co-cultured with *T. gondii* tachyzoites for 24 h, and IL-18 secretion was detected via ELISA (**B**). *n* = 3 per group. **C** and **D**, SARM tuned NLRP3 signal pathway-related proteins in HT22 cells and BV2 cells. HT22 cells were transiently transfected with 4 μg of SARM plasmids after 24 h of overexpression of SARM and co-cultured with *T. gondii* tachyzoites for 24 h, and NLRP3 inflammasome signal pathway-related proteins were investigated by western blot (**C**). HT22 cells were stably transfected with SARM and co-cultured with *T. gondii* tachyzoites for 24 h, and NLRP3 signal pathway-related proteins were investigated by western blot (**D**). BV2 cells were transiently transfected with 4 μg of SARM plasmids, after overexpression of SARM 24 h and co-cultured with *T. gondii* tachyzoites for 24 h;, andNLRP3 inflammasome signal pathway-related proteins were investigated by western blot (**E**). *n* = 3 per group. Data are shown as mean ± SD. ns, not significant; *P < 0.05; **P < 0.01; ***P < 0.001

Given the role of SARM in regulating the NLRP3 inflammasome signaling pathway [18], we detected the effect of SARM on IL-18 secretion in *T. gondii* infection via ELISA. The stable transfection of SARM in HT22 cells significantly downregulated intracellular IL-18 secretion after *T. gondii* infection, while transient transfection of SARM in BV2 cells significantly upregulated intracellular IL-18 secretion after *T. gondii* infection (Fig. 7A, B). Then, western blot was used to examine the expression of intracellular Caspase1, NLRP3, IL-18, and IL-1β proteins. The expression of pro-Caspase1, cleaved Caspase1, NLRP3, IL-18, and secreted IL-1β p17 proteins was downregulated during *T. gondii* infection in both transient and stable transfection of SARM in HT22 cells (Fig. 7C, D). In BV2 cells transiently transfected with SARM, the expression of pro-Caspase1, cleaved Caspase1, NLRP3, IL-18, and secreted IL-1β p17 proteins was significantly upregulated during *T. gondii* infection (Fig. 7E). Thus, SARM had a bidirectional role in regulating the NLRP3 inflammasome signaling pathway during *T. gondii* infection.

## Discussion

The purpose of this study was to explore the role of SARM in *T. gondii* infection. For the first time, we demonstrated that SARM expression was increased in *T. gondii* infection. Then we demonstrated that SARM regulated apoptosis and inflammation in host cells during *T. gondii* infection. Specifically, SARM mediated the upregulation of pro-apoptotic proteins BAX, cleaved Caspase 9, cleaved Caspase 3, ROS, CHOP, and GRP78 and downregulated the expression of anti-apoptotic proteins BCL-2 and p-ERK during *T. gondii* infection. Our findings here highlight SARM as a promising target for *T. gondii* control and therapeutics.

SARM, discovered in 2001, has the functions of regulating tissue development, immunity, and apoptosis, among others [12]. In accordance with prior research, Mukherjee et al. discussed how TLR-induced cell death was mediated by SARM, rather than by the typical MyD88-dependent TLR signaling pathway, and was related to the localization of SARM in mitochondria [15]. Zhu and his colleagues found that the deletion of SARM resulted in high expression of the pro-apoptotic gene XIAP associated factor 1 (XAF1) and intensified the pathogenicity of prions in SARM^−/−^ mice. Additionally, Szretter et al. showed that SARM^−/−^ mice were more susceptible to West Nile virus infection [16]. Consequently, these studies indicated that SARM played a neuroprotective role in the pathogenesis of prions [25]. However, another study found that SARM deletion could inhibit the pathogenicity of the La Crosse virus in vivo, and SARM^−/−^ mice exhibited a higher survival rate after infection [17]. Therefore, we hypothesized that SARM plays different roles in different species.

Mitochondria are central to cell metabolism, producing ATP and numerous biosynthetic intermediates, and are involved in cellular stress responses, including autophagy and apoptosis [26]. It has been reported that SARM regulated apoptosis, primarily by modulating the mitochondrial apoptosis pathway. For instance, the activation of the retinoic acid-inducible gene I-like receptor (RIG-I)-induced mitochondrial antiviral signaling protein (MAVS) is crucial for the induction of type I interferon responses and the inhibition of viral replication [17]. In La Crosse virus infection, MAVS activation upregulates SARM expression. Furthermore, SARM, located in mitochondria, interacts with MAVS and induces oxidative stress and mitochondrial damage, ultimately leading to neuronal death [17]. Additionally, upon influenza virus infection of T cells, SARM reduces the expression of p-ERK, inhibits the expression of the anti-apoptotic protein BCL-XL, upregulates the production of reactive oxygen species, activates apoptosis in CD8^+^ T cells to facilitate T cell clearance, and does not affect the expression of the anti-apoptotic protein BCL-2 [27]. Therefore, we investigated the impact of SARM on the mitochondrial apoptosis pathway during *T. gondii* infection, and our findings revealed that SARM upregulated the expression of pro-apoptotic proteins BAX, cleaved Caspase9, and cleaved Caspase3 while downregulating the expression of the anti-apoptotic protein BCL-2. Consequently, we proposed that SARM induces apoptosis via the mitochondrial apoptosis pathway in *T. gondii* infection.

ER stress is implicated in a variety of common diseases, such as localized ischemia, diabetes, and neurodegenerative diseases. The ER induces apoptosis through the inositol-requiring enzyme type 1 (IRE1)/apoptosis signal regulating kinase-1 (ASK1)/c-Jun N-terminal kinase (JNK) pathway, the Caspase12 kinase pathway, and CHOP/growth arrest-and DNA damage-inducible gene153 (GADD153) pathway [28, 29]. The CHOP pathway plays an important role in apoptosis induced by ER stress resulting from pathogenic microorganism infections, neurological disorders, and cancer. Genes encoding ER chaperone proteins, such as binding immunoglobulin protein (Bip)/GRP78 and GRP94, were upregulated in the ER stress-induced apoptosis pathway, and CHOP were activated [30]. Our results showed that SARM upregulated the expression of GRP78, CHOP, and Caspase12 and the production of ROS during *T. gondii* infection. In CHOP-induced apoptosis pathway, several studies have revealed that CHOP can downregulate the expression of BCL-2, BCL-XL, and MCL-1 and upregulate the expression of BIM, BAK, and BAX [31, 32]. While the highly oxidized state of ER led to the leakage of hydrogen peroxide into the cytoplasm, the production of ROS and a series of apoptosis and inflammation was induced [33–37]. In contrast to CHOP-dependent apoptosis, when Caspase12 was activated, it translocated from the ER to the cytoplasm, directly cleaved pro-Caspase9, and subsequently activated the effector Caspase3 to induce apoptosis [38]. Therefore, we proposed that SARM induces mitochondrial apoptosis via the ER stress pathway during *T. gondii* infection.

The MAPK family, one of the oldest and most evolutionarily conserved serine/threonine protein kinases in eukaryotes, is responsible for intracellular signal transduction and regulation of numerous physiological processes, including gene expression, mitosis, metabolism, cell differentiation, movement, stress response, cell proliferation, apoptosis, and senescence [39, 40]. Here, we demonstrated that SARM inhibited the activation of the intracellular MAPK signaling pathway in *T. gondii* infection. ERK1/2 activation participates in the process of anti-apoptosis by controlling cell proliferation and differentiation [41, 42]. ERK1/2 can exert anti-apoptotic effects by downregulating pro-apoptotic proteins and upregulating anti-apoptotic proteins via transcriptional and post-translational mechanisms [43]. Allan et al. found that ERK could promote apoptosis by phosphorylating Caspase9 at threonine 125 [44]. In our study, we found that SARM inhibited the expression of p-ERK proteins in cells during *T. gondii* infection. On the basis of these studies, we hypothesized that SARM may inhibit cell apoptosis by inhibiting the activation of the MAPK signaling pathway during *T. gondii* infection.

SARM not only regulates apoptosis but also plays a role in immune regulation. In this study, we investigated the effects of SARM on the NLRP3 inflammasome pathway during *T. gondii* infection. The results showed that SARM inhibited the activation of the NLRP3 inflammasome pathway in HT22 cells during *T. gondii* infection, while it promoted the activation of NLRP3 inflammasome signaling pathway in BV2 cells during *T. gondii* infection. Prior studies have noted that the accumulation of inflammasome following infection or injury leads to the release of IL-1β and subsequent cell death. Knockout of the SARM gene in macrophages can reduce the release of inflammasome-dependent cytokines and pyroptosis, resulting in increased IL-1β production but decreased pyroptosis. Conversely, with increased intracellular SARM expression, IL-1β release decreased while pyroptosis increased. SARM can inhibit Caspase1 activation by suppressing NLRP3 inflammasome activation, thereby inhibiting IL-1β release [18]. Pudla et al. found that SARM negatively regulated TLR signaling in *Burkholderia pseudomallei* infection [45], yet other studies have argued that SARM positively regulates TLR signal transduction in neurons and liver. For example, Pan and other studies have found that SARM deletion inhibits the activation of TLR4/7/9 and nuclear factor κB (NF-κB) signaling pathways, thereby reducing the inflammatory response induced by a high-fat diet [46]. We found that both transient and stable transfection of SARM in HT22 cells inhibited the secretion of IL18 and the production of cleaved Caspase1, IL-18, secretedIL-1β p17, and NLRP3 proteins during *T. gondii* infection, while transient transfection of SARM in BV2 cells promoted the secretion of IL-18 and the production of cleaved Caspase1, IL-18, secreted IL-1β p17, and NLRP3 proteins. The differential effects of SARM in HT22 and BV2 cells elucidate the cell-specific modulation of immune responses during *T. gondii* infection. In HT22 cells, the negative regulatory function of SARM on inflammatory processes is indispensable for neuronal viability. Conversely, in BV2 cells, SARM’s proinflammatory activities are essential for initiating immune responses and facilitating pathogen eradication. Achieving a balance between the beneficial and detrimental aspects of neuroinflammatory reactions in the brain is imperative, and SARM appears to be a key factor in maintaining this balance. These observations emphasize the intricate role of SARM in the interplay between host and pathogen and imply that therapeutic interventions targeting SARM must consider the distinct cellular contexts in which it operates.

### Limitation

The current study on SARM’s role in *T. gondii* infection, while providing valuable insights, has several limitations, including: the focus on the RH strain, which may not represent other strains’ effects; the use of HT22 and BV2 cell lines that lack the complexity of in vivo conditions; the absence of SARM knockdown experiments to establish its functional role; and limited in vivo validation in KM mice. Future research should expand to include multiple *T. gondii* strains, utilize primary cell cultures and additional mouse strains for more physiologically relevant data, conduct SARM knockdown studies to clarify its mechanistic involvement, and perform longitudinal analyses to understand the temporal dynamics of SARM’s effects throughout infection. These advancements will enhance the applicability of the findings and contribute to the development of targeted therapeutic strategies for toxoplasmosis.

## Conclusions

We have demonstrated how SARM mediates intrinsic apoptosis and inflammation during *T. gondii* infection. the We have verified that *T. gondii* infection induced high expression of SARM in KM mice, HT22 cells, and BV2 cells. SARM activated host cell apoptosis ththe rough the mitochondrial pathway and ER stress pathway and inhibited MAPK signaling pathway during *T. gondii* infection. SARM participated in the regulation of the inflammatory response through the NLRP3 inflammasome signal pathway during *T. gondii* infection. Therefore, these findings suggest that SARM could be a potential new regulatory factor and drug target for host–pathogen interactions, providing an experimental theoretical basis for the prevention and treatment of *T. gondii* infection.

## Supplementary Information

Additional file 1: Figure S1. SARM is relatively highly expressed in the central nervous system. (A) Western blot analysis of the expression of SARM in different tissues. n=3 mice. (B) Expression of *SARM* throughout the human lifespan. Data from https://hbatlas.org/. (C-D) Gene expression of *Sarm* in Mus musculus (C) and Homo sapiens (D). Data from https://brainrnaseq.org/. Data represent mean ± SD.

Additional file 2: Figure S2. The detailed process of the construction of recombinant plasmid. (A) The construction of SARM plasmid. PCR products of SARM, Lane M1: DNA Marker, Lane 1: PCR identification of SARM, Lane 2: PCR identification of SARM, Lane M2: DNA Marker. (B) Digested products by *EcoR* I and *Xba *I, Lane M1: DNA Marker, Lane 1: Digested products of pRK5 by* EcoR* I and* Xba* I, Lane M3: DNA Marker, Lane 2: Digested products of SARM by *EcoR* I and *Xba* I. (C) Colony PCR, Lane M2: DNA Marker, Lane 1: The products of colony PCR. (D) The BLAST result of SARM gene sequence.

Additional file 3: Figure S3. The identification results of SARM overexpression. (A-B) HT22 cells were transiently transfected with 4μg of SARM plasmids, after overexpression of SARM 24 h, SARM mRNA and protein expression were detected via qPCR (A) and western blot (B). (C-D) HT22 cells were stably transfected with SARM, SARM mRNA and protein expression were detected via qPCR (C) and western blot (D). (E-F) BV2 cells were transiently transfected with 4 μg of SARM plasmids, after overexpression of SARM 24 h, SARM mRNA and protein expression were detected via qPCR (E) and western blot (F). (A) n=4 per group; (B-F) n=3 per group. Data are shown as mean ± SD. ns, not significant; ***p < 0.001.

Additional file 4: Figure S4: The protein expression of SARM was detected after T. gondii infection in vitro at different MOI. (A) The protein expression of SARM was detected in HT22 cells infected with *T. gondii *at different MOI. (B) The expression of SARM was detected in BV2 cells infected with *T. gondii *at different MOI. (C) Statistical analysis of the gray value of the protein in Figure A. (D) Statistical analysis of the gray value of the protein in Figure B. n=3 per group. Data are shown as mean ± SD. *p< 0.05, compare with MOI of zero group; **p < 0.01, compare with MOI of zero group; ***p < 0.001, compare with MOI of zero group.

## Data Availability

No datasets were generated or analyzed during the current study.

## Abbreviations

SARM: Sterile alpha and HEAT/Armadillo motif
TLR: Toll-like receptor
TIR: Toll/interleukin-1 receptor
qPCR: Quantitative real-time polymerase chain reaction
EdU: Ethynyldeoxyuridine
ELISA: Enzyme-linked immunosorbent assay
NLRP3: Nod-like receptor pyrin domain 3
NLRs: Nod-like receptors
IL-18: Interleukin 18
Caspase: Cysteinyl aspartate specific proteinase
CARD: Caspase recruitment domain
TNF-α: Tumor necrosis factor alpha
ROS: Reactive oxygen species
CHOP: C/EBP homologous protein
MAPK: Mitogen-activated protein kinase
GRP78: G-protein coupled receptor 78
MOI: Multiplicity of infection
hpi: H post-infection
dpi: Days post-infection
BCL-2: B-cell lymphoma-2
BAX: BCL2-Associated X BCL-XL BCL-extra large
ERK: Extracellular regulated protein kinases
MAVS: Mitochondrial antiviral signaling protein
PBS: Phosphate-buffered saline
IL-1R: IL-1 Receptor
MIP-1α: Macrophage inflammatory protein-1 alpha
MCP-1: Monocyte chemoattractant protein-1
RANTES: Regulated on activation, normal T cell expressed, and secreted
IFNα: Interferon α
ADAMTS-13: Disintegrin and metalloproteinase with a thrombospondin type 1 motif, member 13
TNFR1: TNF receptor 1
NO: Nitric oxide
ROP18: Polymorphic rhoptry protein 18
RTN1-C: Reticulon 1-C
PVDF: Polyvinylidene fluoride
XAF1: XIAP associated factor 1
ER: Endoplasmic reticulum
RIG-I: Retinoic acid-inducible gene I-like receptor
IRE1: Inositol-requiring enzyme type 1
ASK1: Apoptosis signal regulating kinase-1
JNK: C-Jun N-terminal kinase
GADD153: Growth arrest-and DNA damage-inducible gene153
Bip: Binding immunoglobulin protein
SDS-PAGE: Sodium dodecyl sulfate polyacrylamide gel electrophoresis
DCFH-DA: 2′-7′-Dichlorodihydrofluorescein diacetate
Abs: Antibodies
SD: Standard deviation
DCFH-DA: Antibodies
DCFH-DA: Antibodies
DCFH-DA: Antibodies
