# Supplementary figures and images for: SARM regulates cell apoptosis and inflammation during Toxoplasma gondii infection through a multistep mechanism
Source: Parasit Vectors. 2025 Mar 12;18:103. doi: 10.1186/s13071-025-06721-2 (PMC11899056; doi:10.1186/s13071-025-06721-2)

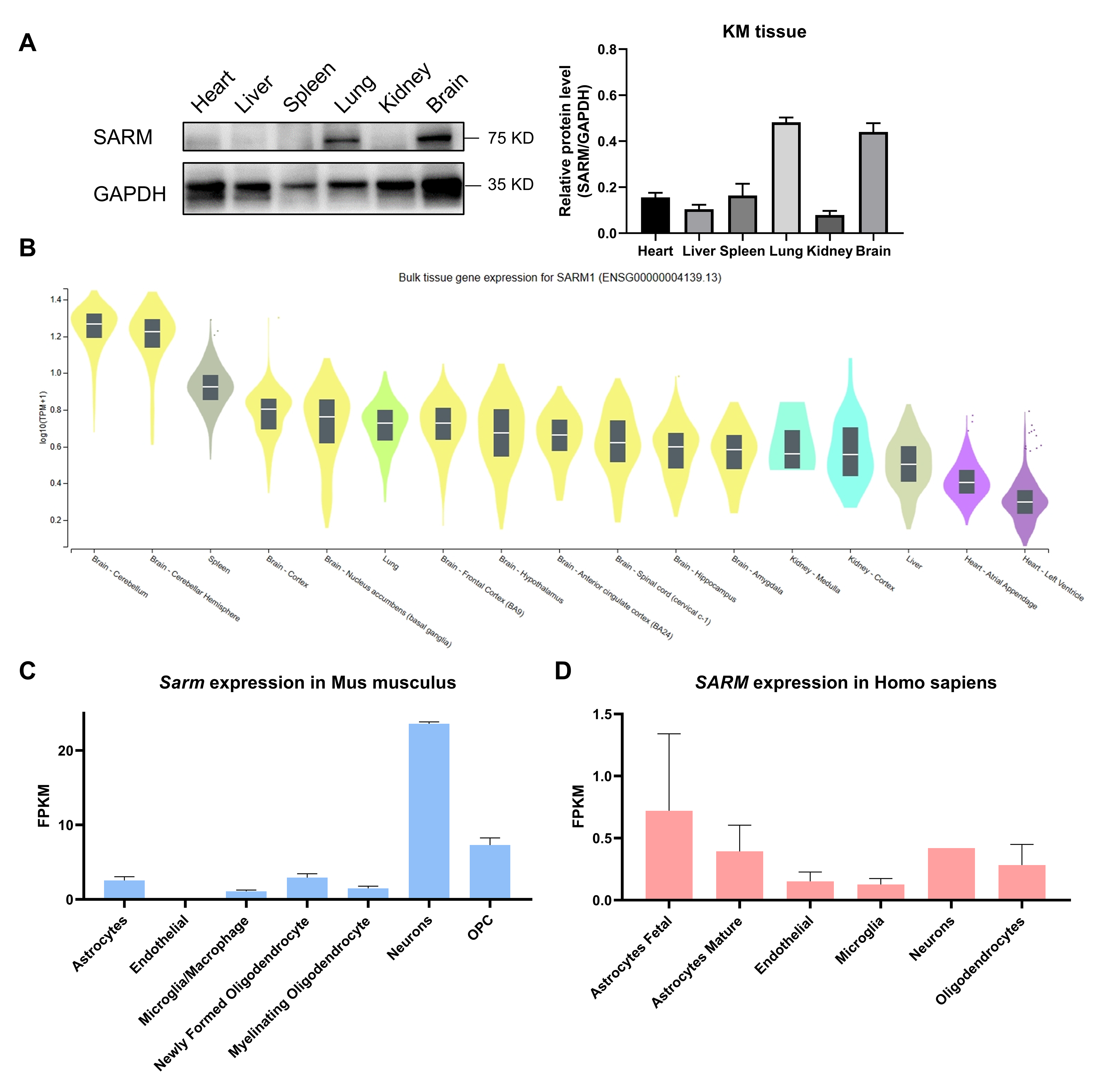

Supplement: Supplementary file 1 — Additional file 1: Figure S1. SARM is relatively highly expressed in the central nervous system. (A) Western blot analysis of the expression of SARM in different tissues. n=3 mice. (B) Expression of SARM throughout the human lifespan. Data from https://hbatlas.org/. (C-D) Gene expression of Sarm in Mus musculus (C) and Homo sapiens (D). Data from https://brainrnaseq.org/. Data represent mean ± SD. [file 13071_2025_6721_MOESM1_ESM.tif]

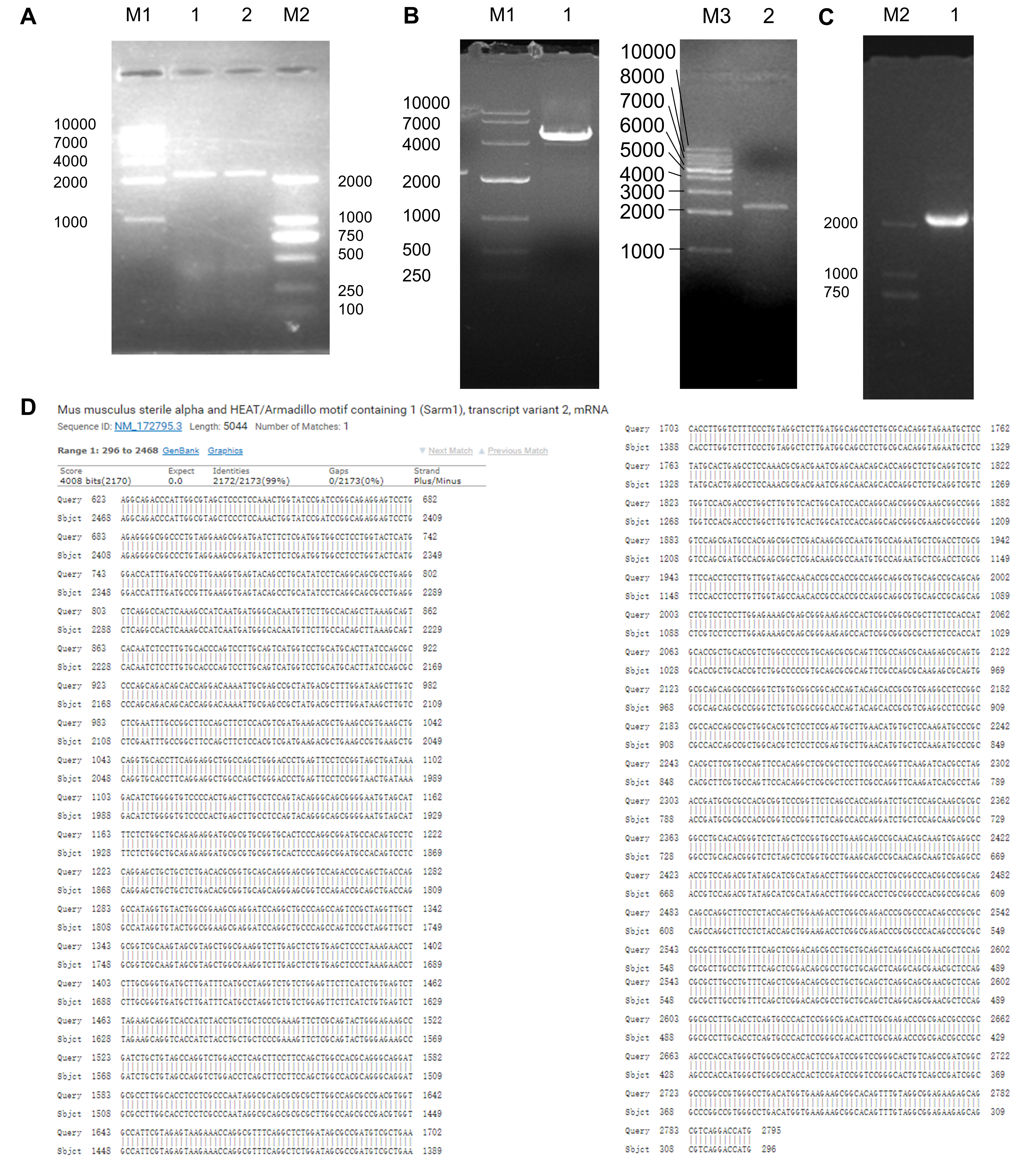

Supplement: Supplementary file 2 — Additional file 2: Figure S2. The detailed process of the construction of recombinant plasmid. (A) The construction of SARM plasmid. PCR products of SARM, Lane M1: DNA Marker, Lane 1: PCR identification of SARM, Lane 2: PCR identification of SARM, Lane M2: DNA Marker. (B) Digested products by EcoR I and Xba I, Lane M1: DNA Marker, Lane 1: Digested products of pRK5 by EcoR I and Xba I, Lane M3: DNA Marker, Lane 2: Digested products of SARM by EcoR I and Xba I. (C) Colony PCR, Lane M2: DNA Marker, Lane 1: The products of colony PCR. (D) The BLAST result of SARM gene sequence. [file 13071_2025_6721_MOESM2_ESM.tif]

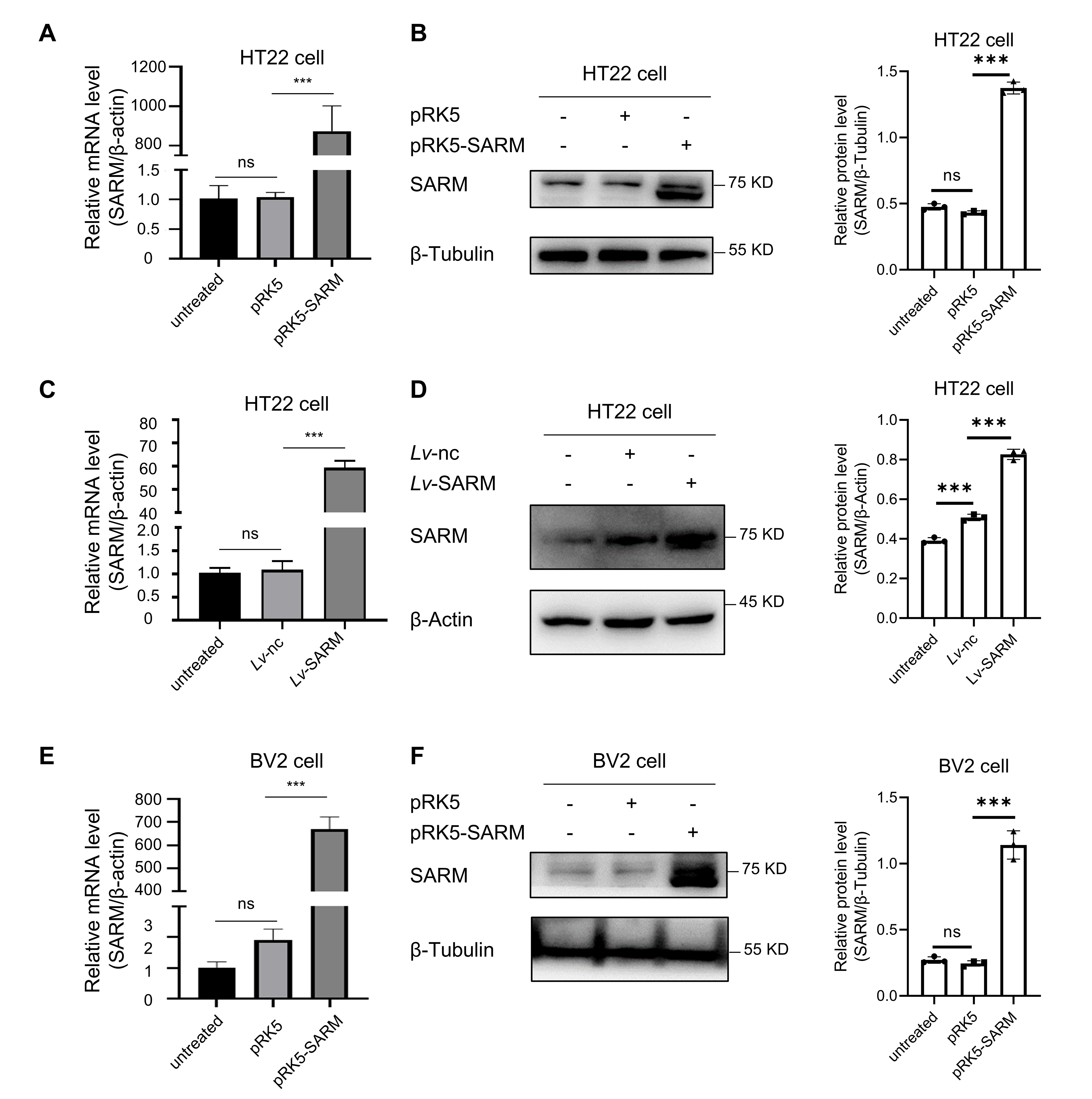

Supplement: Supplementary file 3 — Additional file 3: Figure S3. The identification results of SARM overexpression. (A-B) HT22 cells were transiently transfected with 4μg of SARM plasmids, after overexpression of SARM 24 h, SARM mRNA and protein expression were detected via qPCR (A) and western blot (B). (C-D) HT22 cells were stably transfected with SARM, SARM mRNA and protein expression were detected via qPCR (C) and western blot (D). (E-F) BV2 cells were transiently transfected with 4 μg of SARM plasmids, after overexpression of SARM 24 h, SARM mRNA and protein expression were detected via qPCR (E) and western blot (F). (A) n=4 per group; (B-F) n=3 per group. Data are shown as mean ± SD. ns, not significant; ***p < 0.001. [file 13071_2025_6721_MOESM3_ESM.tif]

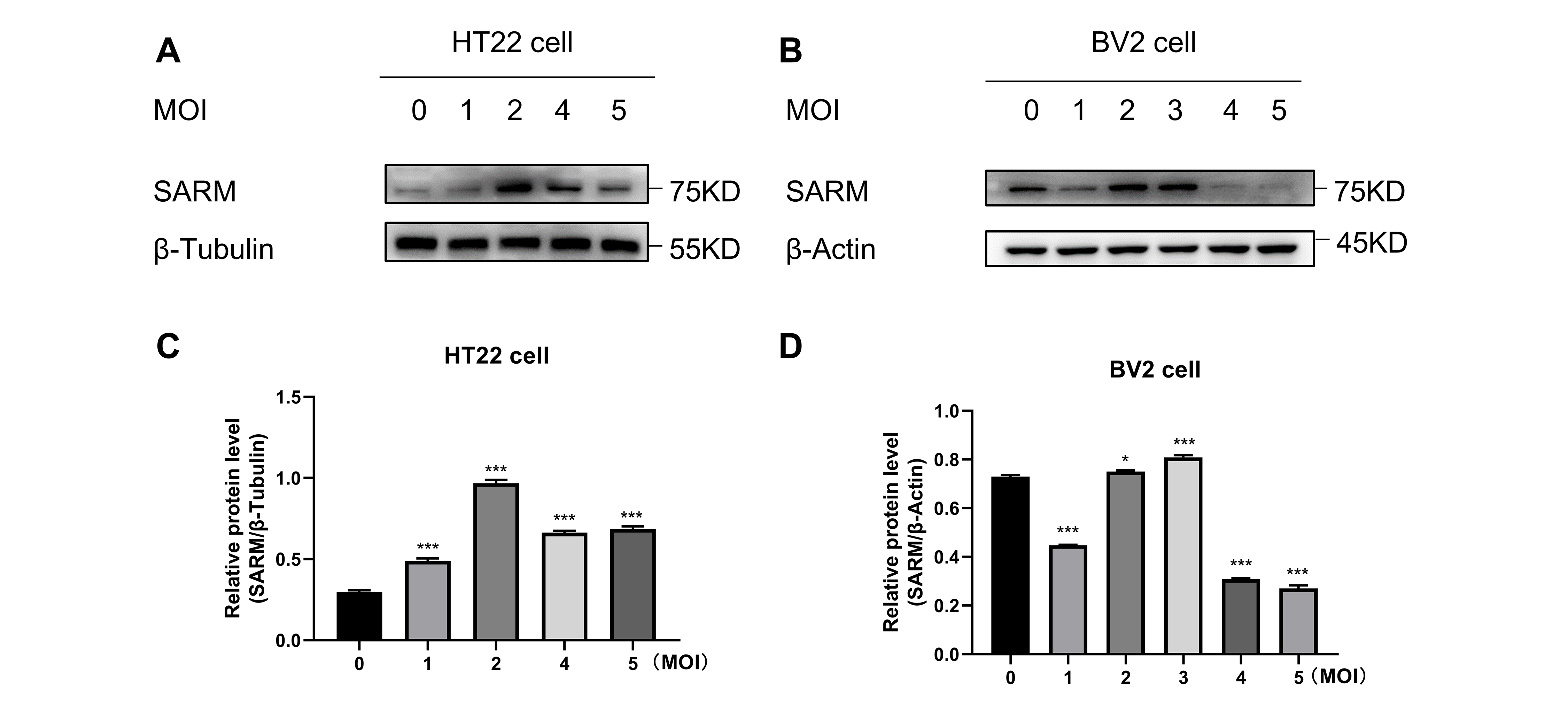

Supplement: Supplementary file 4 — Additional file 4: Figure S4: The protein expression of SARM was detected after T. gondii infection in vitro at different MOI. (A) The protein expression of SARM was detected in HT22 cells infected with T. gondii at different MOI. (B) The expression of SARM was detected in BV2 cells infected with T. gondii at different MOI. (C) Statistical analysis of the gray value of the protein in Figure A. (D) Statistical analysis of the gray value of the protein in Figure B. n=3 per group. Data are shown as mean ± SD. *p< 0.05, compare with MOI of zero group; **p < 0.01, compare with MOI of zero group; ***p < 0.001, compare with MOI of zero group. [file 13071_2025_6721_MOESM4_ESM.tif]
